# Identification of circRNA-miRNA-mRNA network as biomarkers for interstitial cystitis/bladder pain syndrome

**DOI:** 10.18632/aging.205170

**Published:** 2023-11-02

**Authors:** Shi-Qin Yang, Liao Peng, Le-De Lin, Yuan-Zhuo Chen, Meng-Zhu Liu, Chi Zhang, Jia-Wei Chen, De-Yi Luo

**Affiliations:** 1 Department of Urology, Institute of Urology, West China Hospital, Sichuan University, Chengdu, Sichuan 610041, P.R. China

**Keywords:** circRNA, miRNA, circRNA-miRNA-mRNA network, circ.5863, interstitial cystitis

## Abstract

Interstitial cystitis/bladder pain syndrome (IC/BPS) is a long-lasting and incapacitating disease, and the exact factors that affect its onset and advancement are still uncertain. Thus, the main aim was to explore new biomarkers and possible therapeutic targets for IC/BPS. Next-generation high-throughput sequencing experiments were performed on bladder tissues. Based on the interactions between circRNA and miRNA, as well as miRNA and mRNA, candidates were selected to build a network of circRNA-miRNA-mRNA. The STRING database and Cytoscape software were utilized to build a protein-protein interaction (PPI) network to pinpoint the hub genes associated with IC/BPS. The expression levels of circRNA and miRNA in the network were confirmed through quantitative polymerase chain reaction. Western blot was applied to confirm the stability of the lipopolysaccharide-induced IC/BPS model, and the effect of overexpression of circ.5863 by lentivirus on inflammation. Ten circRNA-miRNA interactions involving three circRNAs and six miRNAs were identified, and IFIT3 and RSAD2 were identified as hub genes in the resulting PPI network with 19 nodes. Circ.5863 showed a statistically significant decrease in the constructed model, which is consistent with the sequencing results, and overexpression via lentiviral transfection of circ.5863 was found to alleviate inflammation damage. In this study, a circRNA-miRNA-mRNA network was successfully constructed, and IFIT3 and RSAD2 were identified as hub genes. Our findings suggest that circ.5863 can mitigate inflammation damage in IC/BPS. The identified marker genes may serve as valuable targets for future research aimed at developing diagnostic tools and more effective therapies for IC/BPS.

## INTRODUCTION

Interstitial cystitis/bladder pain syndrome (IC/BPS) represents a long-lasting and incapacitating condition that severely affects the standard of living of those affected, yet its etiology remains unknown [1]. IC/BPS is estimated to affect a percentage ranging from 0.01% to 6.5% of the general population, with a higher incidence in women [2]. Unfortunately, the diagnosis and treatment of IC/BPS are hindered by our limited understanding of its etiology and pathophysiology [3]. Therefore, it is crucial to investigate the molecular mechanisms behind IC/BPS to develop accurate diagnostic criteria and effective therapeutic strategies.

Circular RNA (circRNA) is an endogenous non-coding RNA that possesses a distinctive covalently closed loop structure lacking both a 5′ cap and a poly(A) tail at its 3′ ends [4]. CircRNA is expressed in a variety of mammalian tissues, including serum and tumor tissues [5, 6], and acts as a molecular sponge to effectively bind and suppress miRNA expression [7, 8]. By modulating downstream gene expression through this mechanism, circRNA can exert a pivotal role in the pathogenesis of various diseases [9]. A recent research [10] has shown that circRNA promotes IC/BPS cell proliferation, migration, epithelial-mesenchymal transition (EMT), and inflammation by regulating miRNA expression. Thus, targeting circRNA may represent a promising therapeutic approach for IC/BPS. Nevertheless, the contribution of other circRNAs, miRNAs, and their underlying functions in the development of IC/BPS are still not completely comprehended.

To address this gap in knowledge, we have sequenced bladder samples from IC/BPS patients and non-IC/BPS patients to identify CircRNAs, miRNAs, and mRNAs exhibiting differential expression (DEcircRNAs, DEmiRNAs, and DEmRNAs, respectively). Our aim is to propose a novel diagnostic and therapeutic strategy for IC/BPS, as there are currently only a few studies [10–12] investigating the role of circRNA or miRNA in this condition, and none that have collected samples from IC/BPS patients for high-throughput sequencing.

## RESULTS

### Identification of DEcircRNAs, DEmiRNAs and DEmRNAs

**Figure 1:**
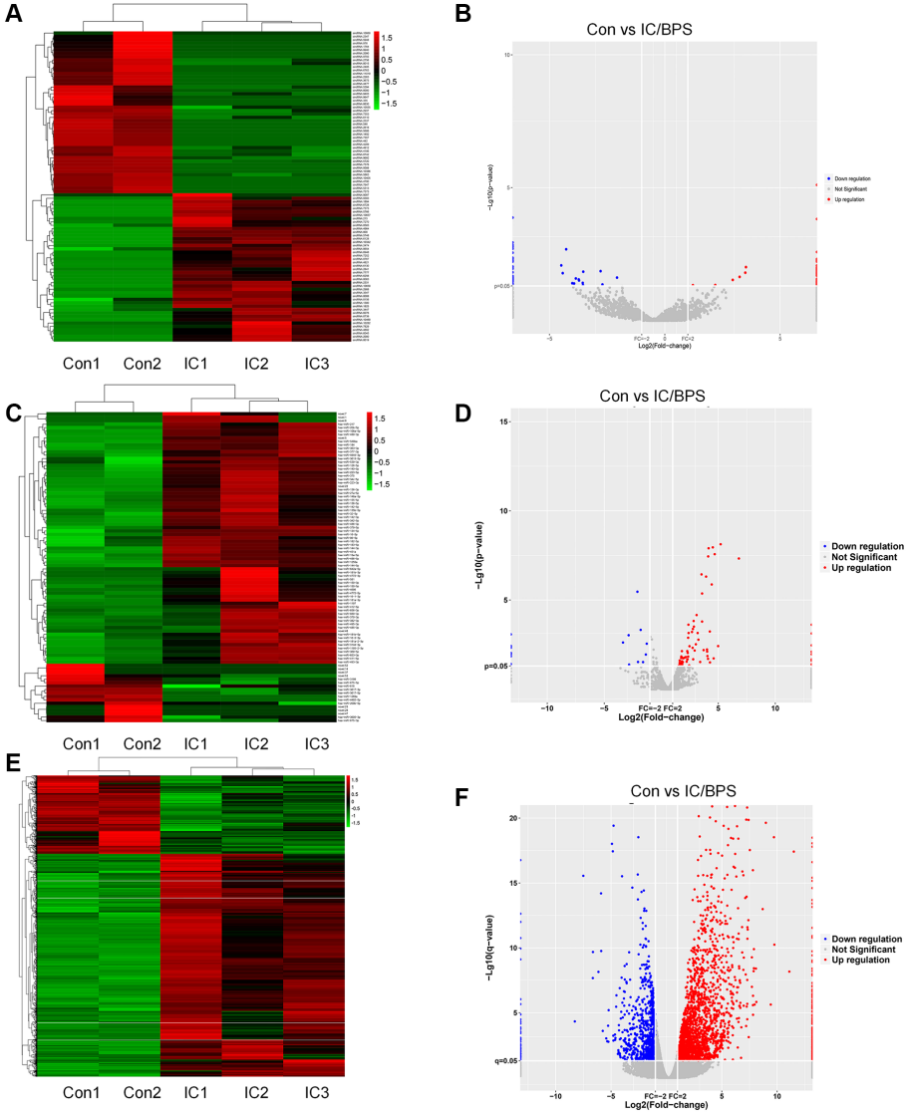
**Heatmaps and volcano plots of DEcircRNAs, DEmiRNAs and DEmRNAs in IC/BPS.** (**A**, **B**) Heatmaps and volcano plots of DEcircRNAs. (**C**, **D**) Heatmaps and volcano plots of DEmiRNAs. (**E**, **F**) Heatmaps and volcano plots of DEmRNAs.

According to the predetermined threshold (|log2FC| ≥ 2.0 and adjusted *P* value < 0.05), comparisons were made between samples from patients with IC/BPS and samples from patients with pure stress urinary incontinence (PSUI). As a result, 94 DEcircRNAs (45 upregulated and 49 downregulated), 92 DEmiRNAs (74 upregulated and 18 downregulated) and 3103 DEmRNAs (2296 upregulated and 807 downregulated) were identified. Subsequently, the heat map and volcano plots representing the DEcircRNAs, DEmiRNAs, and DEmRNAs are depicted in Figure 1.

### GO term and KEGG pathways enrichment analysis

**Figure 2:**
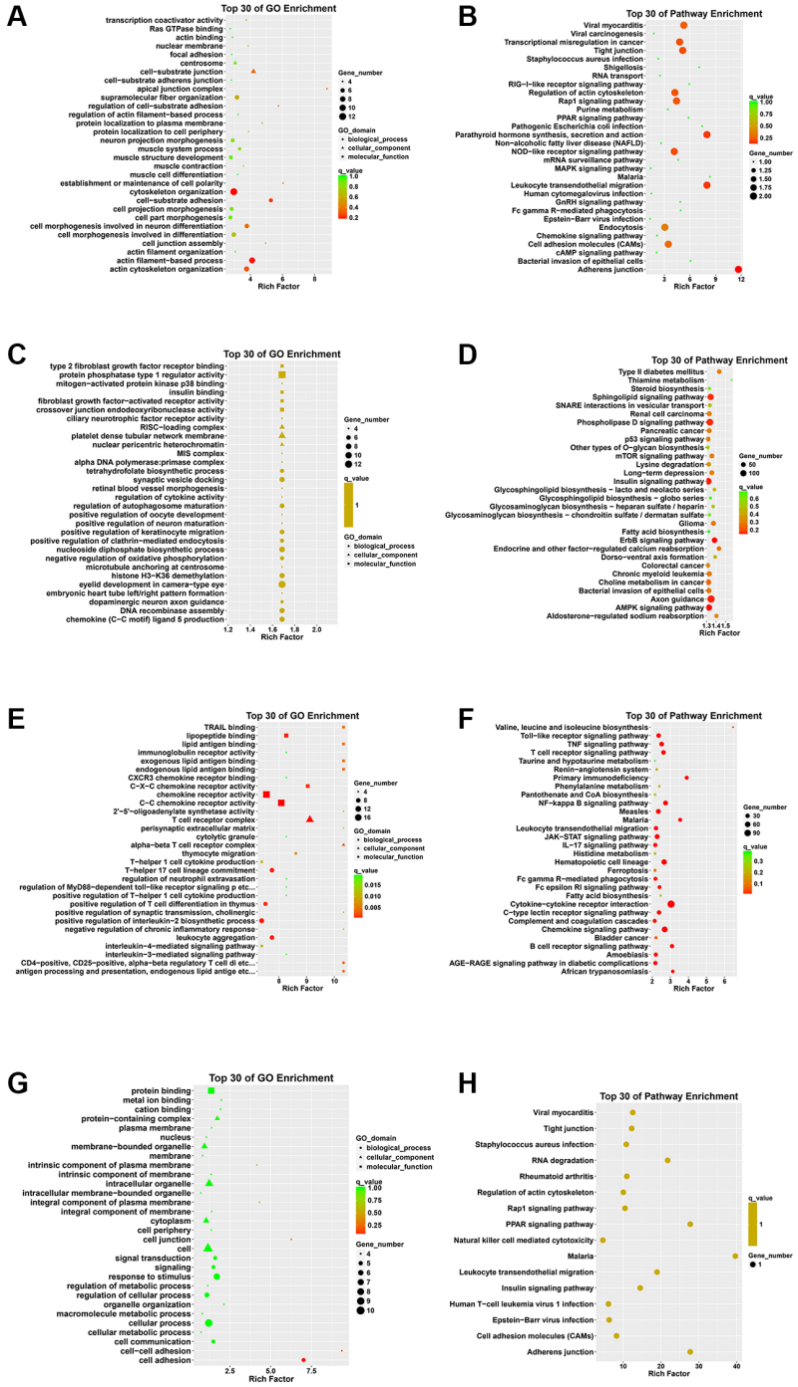
**GO annotation and KEGG pathway analysis of DEcircRNA parental genes, DEmiRNA target genes, DEmRNAs, and genes that intersect DEcircRNA parental genes with DEmRNAs.** (**A**) Top 30 enriched GO terms of the DEcircRNA parental genes. (**B**) Top 30 enriched KEGG pathways of the DEcircRNA parental genes. (**C**) Top 30 enriched GO terms of the DEmiRNA target genes. (**D**) Top 30 enriched KEGG pathways of the DEmiRNA target genes. (**E**) Top 30 enriched GO terms of the DEmRNAs. (**F**) Top 30 enriched KEGG pathways of the DEmRNAs. (**G**) Top 30 enriched GO terms of genes that intersect DEcircRNA parental genes with DEmRNAs. (**H**) Top 30 enriched KEGG pathways of genes that intersect DEcircRNA parental genes with DEmRNAs.

Initially, the results of functional enrichment analysis revealed crucial associations of the DEcircRNAs parental genes with pathways related to the apical junction complex, cell-substrate adhesion, and adherens junction (Figure 2A, 2B).

In addition, GO enrichment analysis of DEmiRNAs target genes revealed that the RISC-loading complex is the main cellular component, and protein phosphatase type 1 regular activity is the main molecular function. Besides, the KEGG pathway analysis demonstrated significant enrichment of the DEmiRNAs in the AMPK signaling pathway (Figure 2C, 2D).

Moreover, the enrichment analysis of the DEmRNAs revealed prominent GO terms such as T cell receptor complex and chemokine receptor activity. Additionally, the KEGG pathway analysis highlighted significant involvement in pathways such as T cell receptor signaling pathway, Toll-like receptor signaling pathway and Cytokine-cytokine receptor interaction (Figure 2E, 2F).

Then, we performed GO analysis on the genes that intersect DEcirRNAs parental gene with the DEmRNAs. Regarding the biological process (BP) aspect, the overlapping genes exhibited significant enrichment in the process of cell adhesion. Cellular processes and response to stimuli were also notable. Additionally, several enriched terms linked to molecular functions, such as protein binding and metal ion binding, were identified. Furthermore, our attention was directed towards the enriched cellular component terms, such as intracellular organelle and cell junction. Moreover, the KEGG pathway analysis revealed significant enrichment of the overlapping genes in adherens junction and tight junction pathways (Figure 2G, 2H).

### Establishment of the circRNA –miRNA –mRNA network

**Figure 3:**
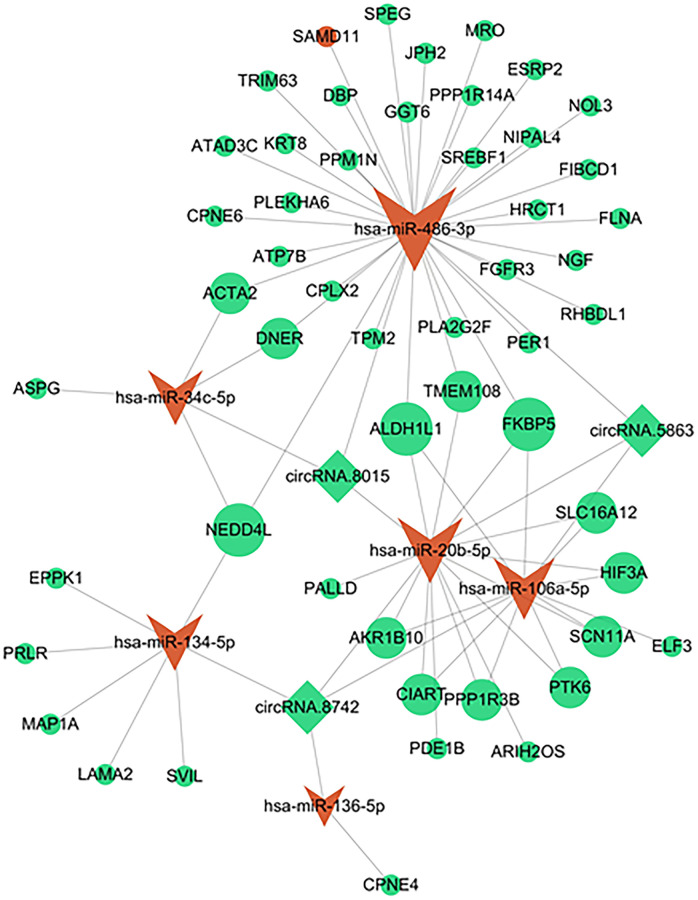
**A circRNA-miRNA-mRNA network in IC/BPS.** The shape of rhombus represents miRNA, V represents miRNAs, and ellipse represents mRNA. Green represents down regulation and red represents up regulation.

The online CircInteractome database was employed to predict the corresponding circRNAs of the DEmiRNAs, enabling the investigation of their possible functional roles in interstitial cystitis. Upon intersecting with the DEcircRNAs, a total of 10 interactions between circRNAs and miRNAs were identified, involving three circRNAs and six miRNAs. By combining the predicted mRNAs of these six miRNAs from the miRanda databases with the DEmRNAs, we obtained a set of 52 target mRNAs. Ultimately, a circRNA-miRNA-mRNA network was constructed by integrating three overlapping circRNAs, six miRNAs, and 52 target mRNAs (Figure 3). Detailed information regarding these three DEcircRNAs can be found in Table 1.

**Table 1:** Basic information of the 3 DEcircRNAs.

**circRNA_id**	**Position**	**Genomic Length**	**Gene symbol**	**Regulation**
circRNA.5863	Chr22:31097269-31104387	7118	SMTN	Down
circRNA.8015	Chr4:38051899-38103157	51258	TBC1D1	Down
circRNA.8742	Chr5:88282042-88292643	10601	TMEM161B-AS1	Down

### Establishment of PPI network and identification of hub genes

**Figure 4:**
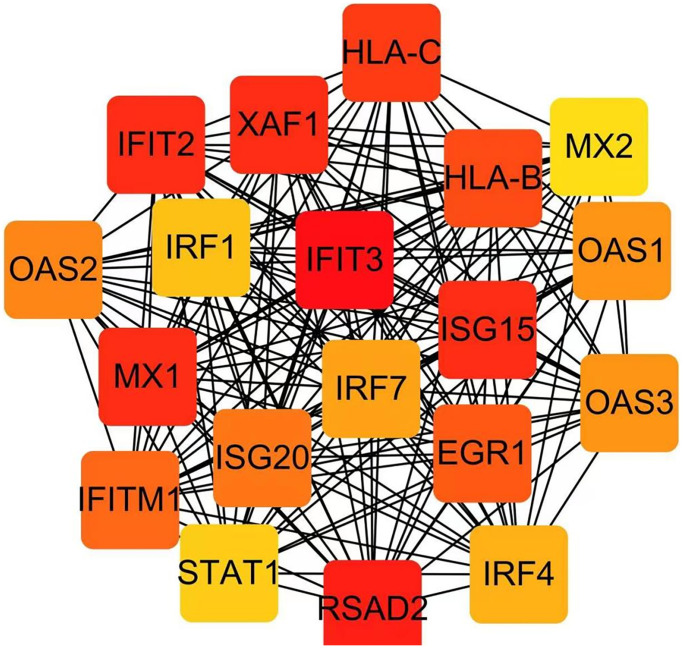
Identification of hub genes in the PPI network with the Cytoscape plugin cytoHubba.

The PPI network, consisting of 19 nodes, was constructed utilizing the STRING database (Figure 4). Subsequently, the cytoHubba app was employed to identify hub genes, resulting in the identification of IFIT3 and RSAD2 as the hub genes.

### Construction of IC/BPS model

**Figure 5:**
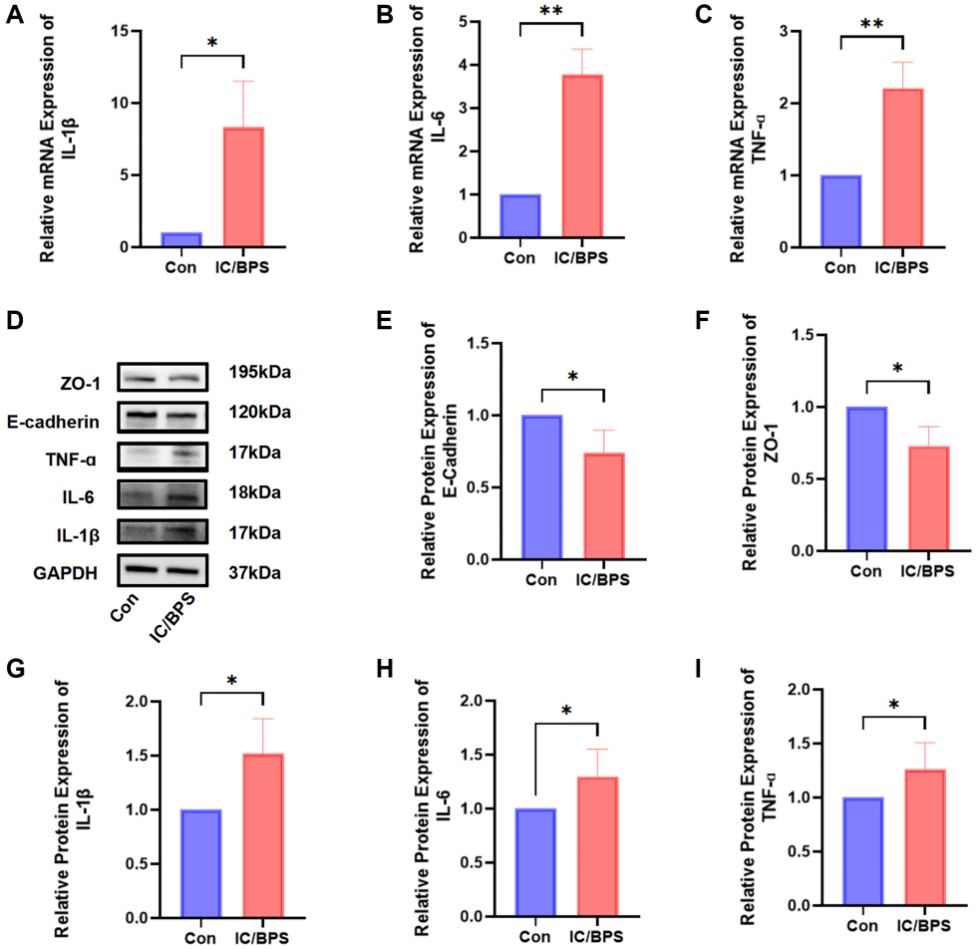
**The construction of an IC/BPS model.** (**A**–**C**) The qPCR showed that the expression levels of IL-1β, IL-6, and TNF-α were significantly higher in the lipopolysaccharide-induced IC/BPS model compared to Con. (**D**) Western blot analysis of ZO-1, E-cadherin, IL-1β, IL-6, TNF-α. (**E**–**I**) Relative protein expression of E-cadherin, ZO-1, IL-1β, IL-6, TNF-α. Results were presented as mean ± SD. ^*^P < 0.05, ^**^P < 0.01. All of the experiments were performed in triplicate.

Following the outlined methodology, an IC/BPS model was established. The Western blotting and q-PCR analyses demonstrated reduced expression levels of barrier function markers (E-Cadherin and ZO-1) and elevated expression levels of inflammatory markers (IL-1β, IL-6, and TNF-α). These results indicate the reliability of our modeling (Figure 5).

### Identification of CircRNA, miRNA in IC/BPS models

**Figure 6:**
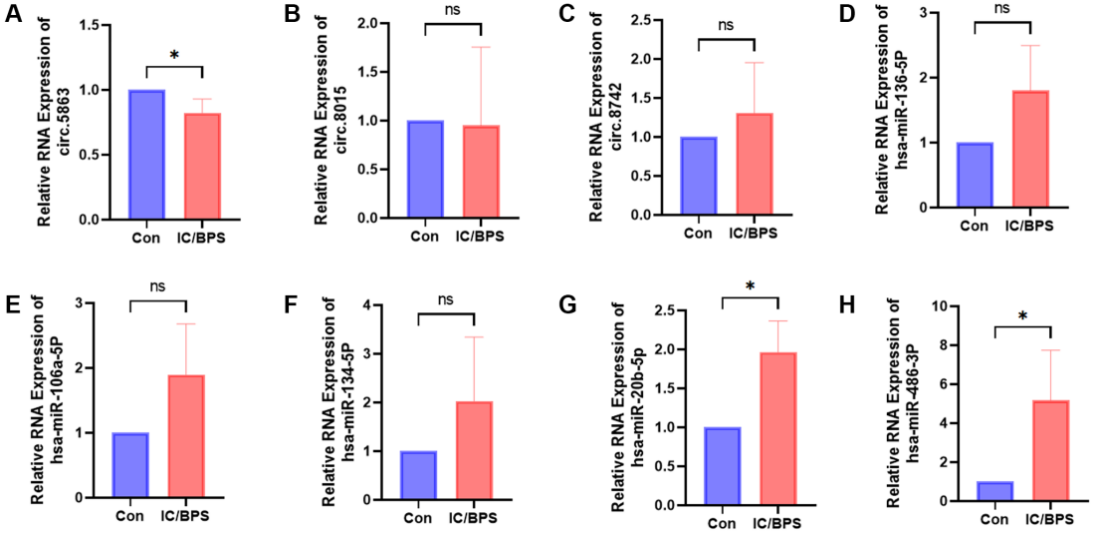
**The relative RNA expression of circRNAs and miRNAs identified in the sequencing-constructed circRNA-miRNA-mRNA network was validated in the lipopolysaccharide-induced IC/BPS model.** (**A**–**H**) The relative RNA expression of circ.5863, circ.8015, circ.8742, hsa-miR-136-5p, hsa-miR-106a-5p, hsa-miR-134-5p, hsa-miR-20b-5p, hsa-miR-486-3p. Results were presented as mean ± SD. ^*^P < 0.05. All of the experiments were performed in triplicate. "Con" refers to the normal HUCs control group, while "IC/BPS" refers to the LPS-induced IC/BPS model group.

To validate our sequencing findings, we performed qPCR validation of the circRNA and miRNA in the network that was constructed in the IC/BPS model. Our qPCR results were in agreement with the sequencing data, demonstrating a significant downregulation of circ.5863 and a significant upregulation of hsa-miR-486-3p and hsa-miR-20b-5p expression levels (Figure 6).

### CircRNA inhibits cell inflammation in HUCs

**Figure 7:**
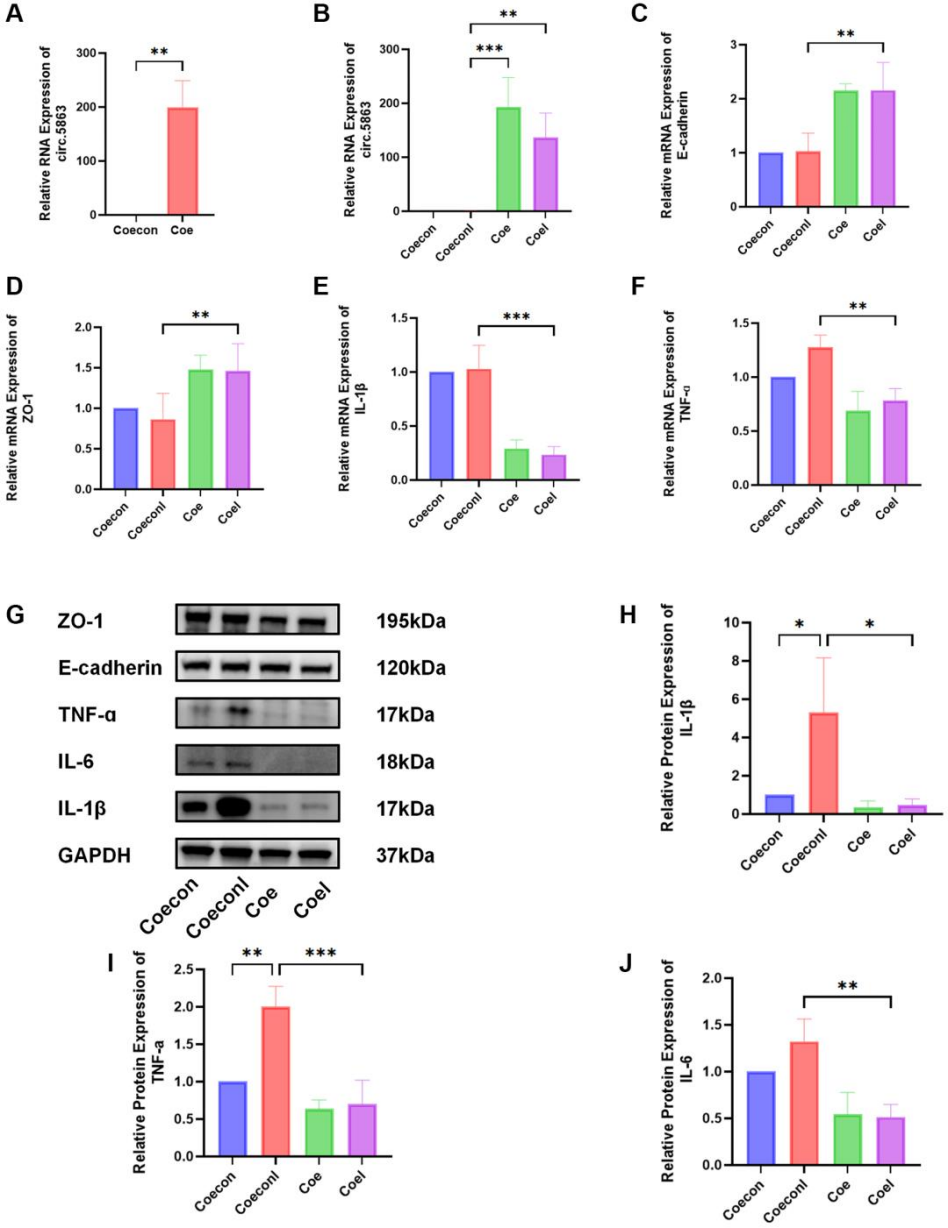
**Overexpression of circ.5863 through lentiviral transfection can alleviate inflammation in IC/BPS.** (**A**) qPCR validation of overexpression of circ.5863. (**B**–**F**) The qPCR results from relative quantitative analysis showed that overexpression of circ.5863 can restore barrier function and reduce the expression levels of IL-1β, and TNF-α. (**G**–**J**) The Western blot results and relative protein quantification analysis showed that overexpression of circ.5863 can reduce the levels of IL-1β, IL-6, and TNF-α. Results were presented as mean ± SD. ^*^P < 0.05, ^**^P < 0.01, ^***^P < 0.001. All of the experiments were performed in triplicate. “coecon” refers to a negative control of HUCs transfected with lentivirus overexpression. “Coeconl” refers to the LPS-induced IC/BPS model of the negative control. “Coe” refers to the overexpression of Circ.5863, while “Coel” refers to the LPS-induced IC/BPS model after overexpression of Circ.5863.

To examine the role of circRNA in the progression of IC/BPS, lentivirus-mediated transduction was employed to achieve stable overexpression of circ.5863 in HUCs. The findings from Western blotting and qPCR suggest that overexpression of circ.5863 can significantly downregulate the levels of IL-6, IL-1β, and TNF-α expression, although there was no significant change in barrier indicators (E-cadherin and ZO-1). The results of both methods are consistent with each other (Figure 7).

## DISCUSSION

IC/BPS is a multifaceted disease with heterogeneous medical presentations and elusive etiology [13]. The precise pathogenesis of IC/BPS remains uncertain, but the aggregation of inflammatory cells and the disruption of barrier function are widely recognized as important pathological features [14]. IC/BPS is currently diagnosed mainly by exclusion, and no complete cure is available. Thus, there is an immediate necessity to search for novel biomarkers for diagnosis and treatment.

CircRNA represents a distinctive class of RNA with a circular structure that makes it more stable and resistant to RNA exonuclease than linear RNA [15]. Researchers have linked circRNA to various diseases, including neurological disorders [16], coronary artery disease [17], and cancers [18], by acting as a competitive endogenous RNA (ceRNA) that competes with miRNAs for binding sites, thereby relieving the suppressive effect of miRNAs on target genes and subsequently upregulating their expression [6]. After validating the sequencing results by qPCR, we observed a significant downregulation of Circ.5853 expression.

Subsequently, we overexpressed Circ.5853 via lentiviral transfection and found that it could reduce inflammation damage and restore barrier function in IC/BPS, as confirmed by qPCR. Western blotting results indicate that overexpression of circ.5863 can significantly downregulate the expression of TNF-α, IL-6, IL-1β, and effectively alleviate inflammation in IC/BPS. According to a previous study [19], the inhibition of miR-20b-5p has been shown to decrease inflammation. Our prediction results indicate a potential interaction between circ.5863 and miR-20b-5p. When circ.5863 is overexpressed, it may function as a molecular sponge for miR-20b-5p, leading to the suppression of miR-20b-5p expression and subsequently reducing the level of inflammation.

MiRNA molecules are small, evolutionarily conserved molecules ranging in length from 18 to 25 nucleotides [20]. Multiple studies have confirmed the link between miRNAs and various diseases, including cancer, diabetes, heart disease, autoimmune disease, and more [6, 20]. In a microarray analysis of miRNA in the GSE11783, Liu et al. identified numerous DEmiRNAs and DEmRNAs between ulcerative IC/PBS and control groups, suggesting their potential involvement in the early detection and progression of IC/BPS [21]. Our qPCR results indicated a statistically significant increase in hsa-miR-486-3P and hsa-miR-20b-5P, which is consistent with the sequencing results, and their stability and tissue specificity suggest their potential as biomarkers for IC/BPS [20]. Interestingly, hsa-miR-486-3p has also been implicated in epithelial-mesenchymal transformation in pulmonary fibrosis, which could be a contributing factor in the pathogenesis of IC/BPS [22]. Their findings highlight the importance of miRNAs in the development of IC/BPS and suggest new avenues for research.

Moreover, IFIT3 plays a vital role in antiviral innate immunity [23], and as human polyomavirus-2 has been detected in the urinary samples of individuals diagnosed with IC/BPS [13], further research is necessary to confirm the significance of IFIT3 in IC/BPS. Overall, our findings shed light on potential mechanisms underlying IC/BPS and suggest promising avenues for future research.

While our study sheds light on the circRNA-miRNA-mRNA network of IC/BPS, it is important to acknowledge its limitations. The sample size was not extensive, and exploring a wider range of molecules is crucial. Additionally, bioinformatics analysis alone cannot provide complete insights, animal experiments and a more in-depth investigation of the specific mechanism of circ.5863 are necessary to confirm the significance of the regulatory network. To better understand IC/BPS and develop effective treatments, future studies should utilize larger sample sizes and more comprehensive molecular profiling.

## CONCLUSION

To summarize, our study successfully established a circRNA-miRNA-mRNA network, identified IFIT3 and RSAD2 as hub genes, and found that circ.5863 can reduce inflammation damage in IC/BPS. Investigating the downstream molecular functions and pathways of these genes could reveal potential treatment-related targets and biomarkers for IC/BPS. Further research in this field is imperative to enhance our understanding of the underlying mechanisms of IC/BPS and facilitate the development of efficacious interventions.

## METHODS

### Sample collection

The main aim of this study was to explore new biomarkers and possible therapeutic targets for IC/BPS. Human research was approved by the Medical Ethics Committee of West China Hospital, Sichuan University (#2019186). Three IC/BPS patients who underwent cystectomy at the Department of Urology of West China Hospital at Sichuan University from July 2018 to December 2019 were included in the study. The two control patients comprised female patients with a diagnosis of pure stress urinary incontinence (PSUI) and stable bladder function, who were admitted for anti-incontinence surgery. During the assessment of bladder injuries, control samples were obtained from these patients by performing transurethral resection of the bladder after sling procedures. Full-thickness bladder tissues were collected from five participants, immediately frozen, and preserved at −80 °C until further use. Supplementary Table 1 presents the inclusion and exclusion criteria utilized for IC/BPS patients. Supplementary Table 2 presents the characteristics of three IC/BPS patients and two PSUI patients.

### Constructing sample library for sequence

A miRNA extraction kit (Cat #TR205-200, Tanmo) was utilized to extract total RNA from the collected tissues in accordance with the guidelines provided by the manufacturer. The quality of RNA was determined using an Agilent Bioanalyzer 2100 (Agilent Technologies, Santa Clara, CA, USA) to obtain a RIN number. The qualified total RNA was purified with the RNAClean XP Kit (Cat A63987, Beckman Coulter, Inc., Brea, CA, USA) and treated with the RNase-Free DNase Set (Cat #79254, QIAGEN, Venlo, The Netherlands) to remove any trace of genomic DNA. After purification, the RNA underwent a series of steps, including rRNA removal, fragmentation, first-strand cDNA synthesis, second-strand cDNA synthesis, end repair, 3′ end plus A, ligation linker, enrichment, and other processes, to create the sequencing sample library.

### Sequencing the constructed library of cDNA

Next Generation Sequencing technology (Illumina Hiseq 2000/2500, Miseq) was used by Shanghai Bohao Biotechnology to sequence cDNA, followed by base identification and error filtering to obtain clean reads. Then, seqtk was utilized to process the clean reads and remove unqualified reads with sequencing primers, low sequencing quality, and low-end quality.

### Identification of DECircRNAs

After completing the previous steps, the reference genome (GRCh38) was aligned using BWA-MEM [24] with default settings for the reads to perform further analysis, and CIRI [25] was employed to predict circRNAs. To differentiate between novel and known circRNAs based on their position information, the identified circRNAs were compared to the circBase (http://circrna.org/) database. By applying the thresholds of |log2(fold-change)| ≥2 and adjusted *P*-value ≤ 0.05, EdgeR [26] analysis was conducted to determine the DEcircRNAs.

### Identification of DEmiRNAs

The constructed sequencing sample library was sequenced on the Illumina HiSeq machine to obtain raw reads, which were subsequently filtered to obtain clean reads suitable for data analysis. Fastx (version: 0.0.13) was utilized to remove unqualified reads. Subsequently, Bowtie [27] was used to compare the clean reads ranging from 18–40 nt to the GRCh38 to identify miRNAs and other small RNAs based on the position information of known miRNAs in miRBase [28] and other ncRNAs. The sRNA Toolkit software package [29] was utilized to predict novel miRNAs. By applying the thresholds of |log2(fold-change)| ≥2 and adjusted *P*-value ≤ 0.05, EdgeR analysis was conducted to determine the DEmiRNAs.

### Identification of DEmRNAs

After constructing the sequencing library, the spliced mapping algorithm of Hisat2 (version: 2.0.4) [30] was utilized to map clean reads to the GRCh38 genome. The reads were then normalized for gene expression analysis through conversion to Fragments Per Kilobase of exon model per Million mapped (FPKM) reads, as described in [31]. By applying the thresholds of |log2(fold-change)| ≥2 and adjusted *P*-value ≤ 0.05, EdgeR analysis was conducted to determine the DEmRNAs.

### GO and KEGG pathway analysis

Protein-coding genes that match the genome location of DEcircRNAs, referred to as DEcircRNAs parental genes, were obtained based on the position information of the circRNAs. Furthermore, we predicted the genes targeted by the DEmiRNAs by analyzing the binding of DEmiRNAs to the 3′ untranslated regions of mRNA using miRanda (http://www.mirdb.org/) [32]. To evaluate functional enrichment, Gene Ontology (GO) analysis and Kyoto Encyclopedia of Genes and Genomes (KEGG) pathway analysis were utilized using the Database for Annotation, Visualization, and Integration Discovery (DAVID) on the parental genes of DEcircRNAs, target genes of DEmiRNAs, DEmRNAs, and genes that intersect DEcircRNAs parental gene with DEmRNAs [33]. We set the thresholds at *P* < 0.05 and reported the top 30 enriched pathways.

### Establish a circRNA–miRNA–mRNA network

The miRNAs targeted by DEcircRNAs were predicted through analyzing the circRNA sequencing data obtained using the miRanda website tool (http://www.mirdb.org/) [34].

Our final set of circRNAs was derived from overlapping miRNAs in the miRanda and DEmiRNAs. The interactions between miRNA and mRNA were predicted using miRanda and then intersect with DEmRNAs. The network was established, integrating candidate target mRNAs and intersecting DEcircRNA-predicted miRNAs with corresponding DEmiRNAs. The network was visualized by using Cytoscape software (Version 3.7.1).

### Building the PPI network and pinpointing the hub genes

To unravel the protein-protein interaction (PPI) network of the DEmRNAs, we employed the STRING database, a powerful tool for exploring functional associations between proteins, available at http://string-db.org/ [35]. Cytoscape 3.7.1 and the cytoHubba app was utilized to pinpoint the hub genes, which are considered the key players in the intricate network of interactions.

### Cellular cultivation and transfection

HUCs (human urothelial cells) sourced from the ATCC CRL-9520 cell line were nurtured in F12K (10% fetal calf serum, 100 μg/ml streptomycin, 100 U/ml penicillin, Hyclone, USA) under optimal conditions of 37°C, 95% air, and 5% CO2. The lentiviral vector GV689 (CMV-circRNA-EF1a-ZsGreen1-T2A-puromycin) obtained from Shanghai GeneChem, China was used to insert the synthesized sequences inducing the overexpression of circ.5863 (Supplementary Table 3). The optimal multiplicity of infection (MOI) index was 100 (Supplementary Figure 1).

### Lipopolysaccharide-induced IC/BPS model

In order to construct a cellular model for IC/BPS, HUCs were subjected to incubation with 50 μg/ml of lipopolysaccharide (LPS, L2880) for 24 hours, using a well-established method to induce inflammation [36].

### Quantitative polymerase chain reaction

Bio-Rad CFX Manager^™^ software (version 3.1) with a Bio-Rad CFX96 instrument was used to perform quantitative polymerase chain reaction (qPCR), and the qPCR primers used are listed in Supplementary Table 4. In brief, the QIAGEN kit (No. 74104, No. 1038703) was applied to extract the circRNA, total RNA, and miRNA. cDNAs were synthesized using Vazyme (R323-01-AB, R323-01-AD). Then the cDNA, cyber green (Vazyme, Q712-02-AA), and primers were mixed in the 96-well plate before preparation for qPCR procedure. The 2^−ΔΔCt^ method was used to calculate the relative gene expression, with GAPDH and U6 serving as internal controls. All assays were conducted in triplicate with three technical replicates per sample.

### Western blotting

RIPA Lysis and Extraction Buffer (Beyotime Biotechnology, China) was used to disrupt HUCs to extract proteins. Subsequently, the protein concentration was ascertained utilizing the BCA Protein Assay Kit (Beyotime Biotechnology). Following separation using 4–20% SDS-PAGE, the protein samples were transferred to PVDF membranes (Millipore, Billerica, MA, USA). The 5% BSA Blocking Buffer was then applied to the membrane and incubated at room temperature for 1.5 hours. Subsequently, the membrane was subjected to overnight incubation at 4°C with primary antibodies against IL-1β (1:1000; AF5103, Affinity, USA), ZO-1 (1:1000; AF5145, Affinity), E-cadherin (1:1000; AF0131, Affinity), IL-6 (1:1000; A22222, Abclonal, USA), TNF-α (1:1000; ER1919-22, Huabio, USA), and GAPDH (1:5000; ab181602, Abcam, UK). Following that, the HRP-conjugated secondary antibodies (1:5000) were applied to the membrane and allowed to bind specifically to their corresponding protein targets during a 1.5-hour incubation at room temperature. Enhanced chemiluminescence (Thermo Fisher Scientific, Waltham, MA, USA) was then employed to visualize the proteins, generating a radiant glow upon interaction. The resulting bands were captured and their intensities were quantified using the ChemiDoc MP Imaging System (Bio-Rad, Hercules, CA, USA), enabling assessment of protein abundance or expression levels.

### Statistical analysis

GraphPad Prism (version 9.5) was utilized for statistical analysis, and the results were expressed as mean ± SD. For intergroup comparisons, a two-tailed Student’s *t*-test was employed, while multigroup comparisons were performed using one-way ANOVA. The threshold for statistical significance was defined as *P* < 0.05.

### Data availability statement

The data supporting the finding of this article are available from the corresponding author.

## Supplementary Materials

Supplementary Figure 1

Supplementary Tables
