# Supplementary material for: Identification of circRNA-miRNA-mRNA network as biomarkers for interstitial cystitis/bladder pain syndrome
Source: Aging (Albany NY). 2023 Nov 2;15(21):12155–70. doi: 10.18632/aging.205170 (PMC10683623; doi:10.18632/aging.205170)
Supplement: Supplementary Figure 1 [file aging-15-205170-s001.pdf]

SUPPLEMENTARY FIGURE

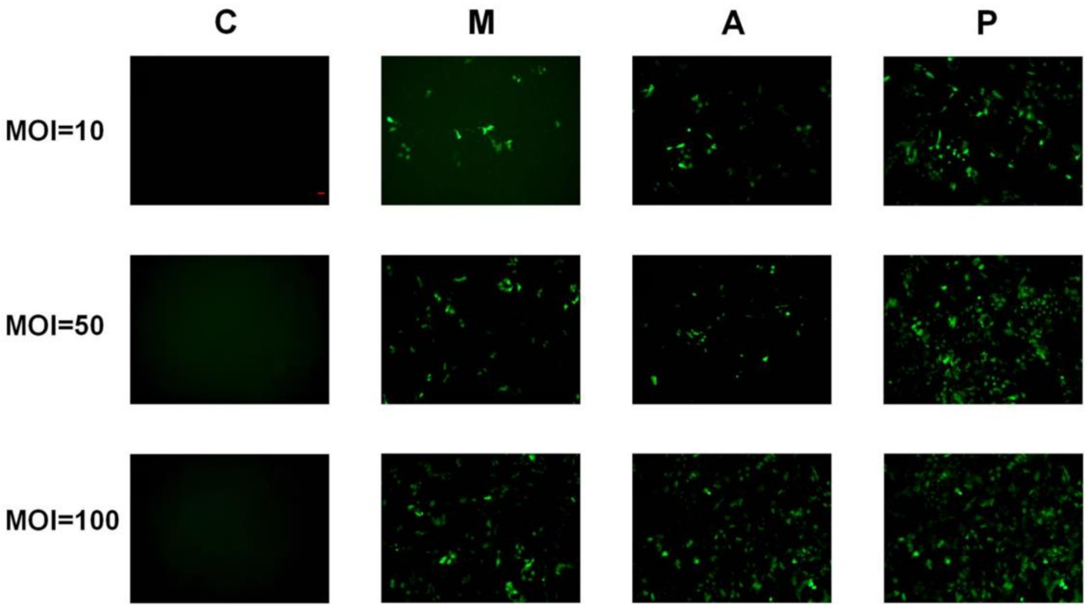

Supplementary Figure 1. Lentiviral transduction. C stands for control group, M stands for transduction with lentivirus only, A stands for transduction with lentivirus and enhanced infection reagent A, P stands for transduction with lentivirus and enhanced infection reagent P.
