# Supplementary material for: Identification of circRNA-miRNA-mRNA network as biomarkers for interstitial cystitis/bladder pain syndrome
Source: Aging (Albany NY). 2023 Nov 2;15(21):12155–70. doi: 10.18632/aging.205170 (PMC10683623; doi:10.18632/aging.205170)
Supplement: Supplementary Tables [file aging-15-205170-s002.pdf]

SUPPLEMENTARY TABLES

Supplementary Table 1. Inclusion and exclusion criteria of the patients with interstitial cystitis.

Inclusion criteria

1. Patients over 18 years old;
2. Previously diagnosed of interstitial cystitis more than 6 months;
3. The characteristic pathological findings in the bladder wall were identified by cystoscopy;
4. O'Leary-Sant Interstitial Cystitis Symptom and Problem Index score over 18;
5. Patients are aware of the purpose of this study and give fully informed consent;

Exclusion criteria

1. General conditions
   1. Currently diagnosed with cancer, severe heart, lung, liver, kidney, or blood disorder.
   2. Patients who are pregnant or desire to become pregnant.
   3. The investigators assessed that the patient was not suitable for the study.
2. Urological problems
   1. Have previous history of urinary infection (e.g., bacterial cystitis) within 12 weeks;
   2. Currently diagnosed with Urethral diseases (e.g., urethral stone, bladder cancer); Gynaecological diseases (e.g., pelvic organ prolapse) or neurogenic urinary frequency
   3. Patients accompanied by urinary tract symptoms (i.e., bladder pain, urinary frequency and/or urinary urgency)
   4. Have previous history of augmentation cystoplasty or cystectomy;
3. Treatment related
   1. Have history of the following therapies within 24 weeks: Hydrodistension, intravesical laser therapy, intravesical electrical coagulation, transurethral resection, pelvic reconstructive surgery, nerve block or spinal cord stimulation for pain relief;
   2. Received intravesical instillation of drugs within 12 weeks.
   3. Have previous history of chemical compound (such as cyclophosphamide) derived cystitis.

Supplementary Table 2. The characteristics of IC/BPS patients and PSUI patients.

Diagnosis	Age (years)	BMI	Duration (months)	Menopause	Hypertension	DM	Voids/ day	Night urination	ICSI	ICPI	VAS
IC/BPS-1	58	23.44	11.0	Post	0	0	20	4	18	16	9
IC/BPS-2	56	23.35	52.0	Post	0	0	21	5	17	14	8
IC/BPS-3	60	22.22	24.0	Post	0	0	21	9	16	15	3
PSUI-1	67	19.25	–	Post	0	0	5	0	0	0	0
PSUI-2	68	24.51	–	Post	0	0	6	0	0	0	0

Abbreviations: BMI: body mass index (kg/m²); DM: diabetes mellitus; IC: interstitial cystitis/bladder pain syndrome; PSUI: pure stress urinary incontinence; ICSI: ICPI interstitial cystitis symptom index and problem index; VAS: visual analogue scale.

Supplementary Table 3. The sequence of circ.5863 transferred by transfection.

circRNA.5863

AAAAAGACCTTGGGGTAGAGGGGCGCCACGAGAGGCGGGTCTGGCGCTGACATCCAACCCCGTGCCCCCTCC
CTGCAGGATGCTGGTGGACTGTGTGCCCCTGGTGGAGGTGGACGACATGATGATCATGGGCAAGAAGCCTGA
CCCCAA*ATGGCAGTGGCAGCACCATGATGCAAACCAAGACCTTCTCCTCTTCCTCCTCATCCAAGAAGATGG
GCAGGTGAGCACCAGCACCCAATCCCTGACCATAGAGGAGTCAGTGCCACAGGGGACCTAACTGCAAACCCG
TTTTACAGAGGG

Supplementary Table 4. Sequence information of primers.

Primers	Sequences (5′–3′)
TNF-α	F: GCTGCACTTTGGAGTGATCG<br>R: GCTTGAGGGTTTGCTACAACA
IL-1β	F: AGCTCGCCAGTGAAATGATG<br>R: GACATGGAGAACACCACTTGT
GAPDH	F: ACAACTTTGGTATCGTGGAAGG<br>R: GCCATCACGCCACAGTTTC
ZO-1	F: CAACATACAGTGACGCTTCACA<br>R: CACTATTGACGTTTCCCCACTC
E-Cadherin	F: GGATGTGAATGAAGCCCCCA<br>R: AGTGGAAATGGCACCAGTGT
IL-6	F: CCTGAACCTTCCAAAGATGGC<br>R: TTCACCAGGCAAGTCTCCTCA
Circ.5863	F: ATCCAAGAAGATGGGCAGGTG<br>R: GGGTTTGCAGTTAGGTCCCC
Circ.8045	F: GGGAATGCGTCATCACCGAG<br>R: TGTATGGCACATCCTTTGGCT
Circ.8742	F: AGGTCTGGAAAGCATGGAAAT<br>R: AGTTCAAACATGAAAAGAGTCCAT
miR-134-5p	F: GCCGAGGGGGAGACCAGUUG<br>R: CTCAACTGGTGTCGTGGA
miR-136-5p	F: ACTCCATTTGTTTTGATGATGGA<br>R: GCAGGGTCCGAGGTATTC
miR-20b-5p	F: CGCCATCAAAGTGCTCATAGTGC<br>R: ATCCAGTGCAGGGTCCGAGG
miR-106a-5p	F: TGCAGTAGATCTCAAAAAGCTACC<br>R: CCTTGGCCATGTAAAAGTGC
miR-486-3p	F: GGGGCAGCTCAGTACA<br>R: GGTCCAGTTTTTTTTTTTTTTTTTTTTTTTTTACCT
U6	F: CTCGCTTCGGCAGCACA<br>R: AACGCTTCACGAATTTGCGT
